# Identification of pH-sensing Sites in the Light Harvesting Complex Stress-related 3 Protein Essential for Triggering Non-photochemical Quenching in Chlamydomonas reinhardtii*

**DOI:** 10.1074/jbc.M115.704601

**Published:** 2016-01-27

**Authors:** Matteo Ballottari, Thuy B. Truong, Eleonora De Re, Erika Erickson, Giulio R. Stella, Graham R. Fleming, Roberto Bassi, Krishna K. Niyogi

**Affiliations:** From the ‡ Department of Biotechnology, University of Verona, Strada Le Grazie, I-37134 Verona, Italy,; the § Howard Hughes Medical Institute, Department of Plant and Microbial Biology, University of California, Berkeley, California 94720-3102,; the ‡‡ Department of Chemistry, Hildebrand B77, University of California, Berkeley, California 94720-1460,; the ** Sorbonne Universités, UPMC Univ-Paris 6, CNRS, UMR 7238, Laboratoire de Biologie Computationnelle et Quantitative, 15 rue de l'Ecole de Médecine, 75006 Paris, France,; the ¶ Molecular Biophysics and Integrated Bioimaging Division, Lawrence Berkeley National Laboratory, Berkeley, California 94720, and; the ‖ Graduate Group in Applied Science and Technology, University of California, Berkeley, California 94720

**Keywords:** fluorescence, photosynthesis, photosynthetic pigment, photosystem II, plant biochemistry, non-photochemical quenching, photoprotection

## Abstract

Light harvesting complex stress-related 3 (LHCSR3) is the protein essential for photoprotective excess energy dissipation (non-photochemical quenching, NPQ) in the model green alga *Chlamydomonas reinhardtii*. Activation of NPQ requires low pH in the thylakoid lumen, which is induced in excess light conditions and sensed by lumen-exposed acidic residues. In this work we have used site-specific mutagenesis *in vivo* and *in vitro* for identification of the residues in LHCSR3 that are responsible for sensing lumen pH. Lumen-exposed protonatable residues, aspartate and glutamate, were mutated to asparagine and glutamine, respectively. By expression in a mutant lacking all LHCSR isoforms, residues Asp^117^, Glu^221^, and Glu^224^ were shown to be essential for LHCSR3-dependent NPQ induction in *C. reinhardtii*. Analysis of recombinant proteins carrying the same mutations refolded *in vitro* with pigments showed that the capacity of responding to low pH by decreasing the fluorescence lifetime, present in the wild-type protein, was lost. Consistent with a role in pH sensing, the mutations led to a substantial reduction in binding the NPQ inhibitor dicyclohexylcarbodiimide.

## Introduction

Photosynthetic organisms convert sunlight absorbed by chlorophyll into chemical energy by reducing CO_2_ into sugars with electrons extracted from water, yielding O_2_. However, molecular oxygen can react with chlorophyll triplets (^3^Chl*)^6^ to yield singlet oxygen, one of several types of reactive oxygen species, which damage biological molecules (1). Because ^3^Chl* originates from ^1^Chl*, prevention of photooxidative stress can be obtained by quenching ^3^Chl*, scavenging reactive oxygen species, or by quenching ^1^Chl*. In addition, the photon absorption rate can be regulated by chloroplast relocation within the cell or changes in leaf orientation (1–3).

Of particular importance are the non-photochemical quenching (NPQ) mechanisms that quench ^1^Chl* and dissipate excess excitation energy as heat when light absorption exceeds the capacity of photochemical reactions. NPQ includes several components, the major and fastest of which is energy-dependent quenching (qE), which is sensitive to uncouplers (4, 5). qE is a feedback process triggered by thylakoid lumen acidification (4, 6–10). Saturation of downstream reactions leads to depletion of ADP and P_i_, the substrates of ATPase, which prevents the efflux of protons generated by photosynthetic electron transport from the thylakoid lumen to the stroma, leading to lumen acidification.

Genetic analysis of qE activation led to the identification of PSBS and LHCSR (11–15) as gene products required for qE in the model plant *Arabidopsis thaliana* and the green alga *Chlamydomonas reinhardtii*, respectively (12, 13, 16–18). In *C. reinhardtii*, two LHCSR isoforms, LHCSR1 and LHCSR3, are active in qE. LHCSR3 strongly accumulates in excess light, whereas LHCSR1 is constitutively present even at low light levels (13, 19). Also, LHCSR-like proteins with qE activity have been identified in diatoms (20–23). A special case is found in mosses, where both PSBS and LHCSR proteins are present and involved in qE induction (15, 24, 25). Although the fundamental mechanisms of quenching activity by PSBS and LHCSR are the subjects of intense investigation (26), they must be different because LHCSR is a chlorophyll- and xanthophyll-binding protein where quenching of ^1^Chl* can be catalyzed as shown by its short fluorescence lifetime (18). In contrast, pigment-binding sites are not conserved in PSBS, suggesting that the quenching activity is elicited within interacting proteins (18, 25, 27). As for the capacity for sensing the lumenal pH, PSBS and LHCSR share the property of binding dicyclohexylcarbodiimide (DCCD), a protein-modifying agent that covalently binds to acidic residues involved in reversible protonation events (28). Indeed, we have previously shown that two glutamate residues in PSBS are responsible for both the DCCD binding *in vitro* and the NPQ activity *in vivo* (17). Sequence analysis of LHCSR proteins showed multiple conserved acidic residues exposed to the lumen as potential sites of protonation. Recombinant LHCSR3 from *C. reinhardtii* has been shown to be pH responsive and to undergo a switch to a dissipative state in acidic solution (18, 27). Mutation analysis has located eight putative pH-sensing residues in the C terminus of LHCSR3 (27), whereas PSII supercomplexes containing LHCSR3 with a stoichiometry LHCSR3: PSII of 0.28 were reported to undergo a decrease in fluorescence lifetime when exposed to pH 5 (29).

Here, we have performed a detailed investigation of the pH-sensing activity in LHCSR3 from *C. reinhardtii*, including identification of lumen-exposed protonatable residues that have been mutated to non-protonatable ones. The effect of these mutations has been analyzed both by fluorescence lifetime analysis of the proteins refolded *in vitro* and by measuring NPQ activity *in vivo* upon expression in a mutant lacking both LHCSR3 and LHCSR1. This comprehensive procedure led to the identification of three residues that are crucial for pH-dependent quenching *in vivo* and *in vitro* and are also responsible, to a large extent, for the binding of the qE inhibitor DCCD.

## Experimental Procedures

### 

#### 

##### LHCSR3 Structure Modeling

LHCSR3 protein structures were obtained using homology modeling techniques with the on-line servers I-TASSER (30, 31) version 1.1. The model with the best C-score (confidence score) was selected for further analysis.

##### Site-directed Mutagenesis of Acidic Residues

The *LHCSR3.1* genomic clone plasmid LHCSR3/GwypBC1 from previous complementation experiments (13) was used for site-directed mutagenesis of each acidic residue reported in the text. The QuikChange® Site-directed Mutagenesis Kit was used according to the manufacturer's instructions.

##### Transformation and Isolation of Site-directed Mutants

The plasmid LHCSR3/GwypBC1 containing each or multiple mutations was transformed into either *npq4* (13) or *npq4 lhcsr1* (32) and transformants were selected for paromomycin resistance. At least 300 colonies were picked for each line and patched onto HS minimal medium to grow in high light (400 μmol of photons m^−2^ s^−1^). NPQ was measured by chlorophyll fluorescence video imaging (Imaging-PAM, Walz). Selected colonies, as judged by their NPQ value relative to the parent strain, were further cultured in liquid HS to measure via a pulse-amplitude-modulated fluorometer (FMS2, Hansatech) and for immunoblots, as previously described (13).

##### Recombinant Protein Overexpression, Purification, and in Vitro Refolding

LHCSR3 coding sequence was cloned in pET28 expression vector and expressed in *Escherichia coli* as previously described (18). Purified apoprotein was refolded *in vitro* in the presence of pigments as reported in Refs. 18 and 33.

##### Pigment Analysis

Pigments bound by recombinant LHCSR3 were measured as described in Ref. 34.

##### SDS-PAGE, Coomassie Staining, DCCD Binding, and Western Blot

SDS-PAGE was performed as reported in Ref. 18. SDS-PAGE gel was then stained with Coomassie-R as described in Ref. 35. DCCD binding properties of recombinant LHCSR3 proteins were estimated by incubating the refolded protein with [^14^C]DCCD and subsequent autoradiography evaluation of the binding as previously described (18, 28, 36). Western blot analysis was performed as described in Ref. 13.

##### Fluorescence Lifetime Measurements

Fluorescence decay kinetics were measured on recombinant LHCSR3 protein using a time-correlated Single Photon Counting apparatus similar to the one described in Ref. 10. 150-fs pulses centered at 820 nm are generated by a Ti:Sapphire oscillator (Coherent Mira 900F) at 76 MHz repetition rate. The pulses are frequency-doubled in a 1-mm thick BBO crystal and their repetition rate is reduced by a factor of 8 with a pulse picker (Spectra Physics model 3980). The resulting pulses are centered at 410 nm, with 12-nm bandwidth full width half-maximum, and with an energy at sample of ∼10 pJ/pulse. The fluorescence emitted by the sample passes through a polarizer set at magic angle, followed by either a monochromator (Horiba Jobin-Ivon H-20) or a long-pass filter. The detection system is composed of a MCP/PMT detector (Hamamtsu R3809U), electrically cooled to −30 °C. The detector is connected to a PC computer with a DCC-100 detector control card (Becker-Hickl). The full width half-maximum of the instrument response function is measured to be 45–55 ps. The samples are held in 1- or 2-mm thick quartz cuvettes (Starna Cells), and kept at ∼12 °C during the measurements with a home-built nitrogen cooling system.

##### Steady-state Absorption, Fluorescence, and Circular Dichroism Measurements

Room temperature absorption spectra were recorded using an SLM-Aminco DK2000 spectrophotometer, in 10 mm HEPES, pH 7.5, 0.2 m sucrose, and 0.03% *n*-dodecyl-α-d-maltopyranoside. The wavelength sampling step was 0.4 nm. Fluorescence emission spectra were measured using a Jobin-Yvon Fluoromax-3 device. Circular dichroism (CD) spectra were measured at 10 °C on a Jasco 600 spectropolarimeter using a R7400U-20 photomultiplier tube: samples were in the same solution described for the absorption with an OD of 1 at the maximum in the Qy transition. The measurements were performed in a 1-cm cuvette. Denaturation temperature measurements were performed by following the decay of the CD signal at 682 nm when increasing the temperature from 20 to 80 °C with a time slope of 1 °C/min and a resolution of 0.2 °C. The thermal stability of the samples was determined by finding the *T*_½_ of the signal decay.

##### Dynamic Light Scattering Measurements

The size of aggregates induced by detergent dilution was determined by dynamic light scattering using ZETASIZER NANO S instrumentation as described in Refs. 37 and 38.

## Results

### 

#### 

##### Structural Model of LHCSR3

**FIGURE 1.**
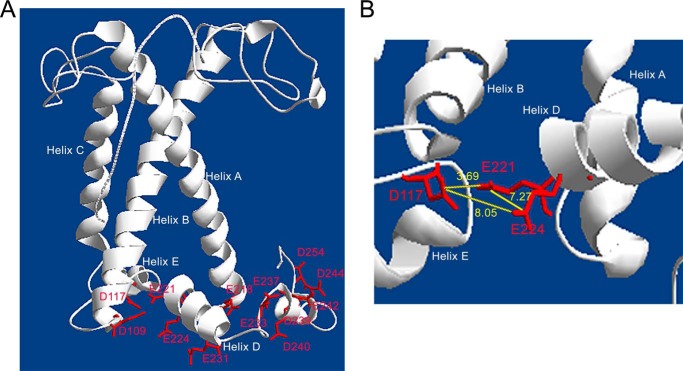
**Three-dimensional model of LHCSR3.** *Panel A,* LHCSR3 structure modeled on LHCII and CP29 crystallographic structures. Putative protonatable sites are indicated. *Panel B,* zoom view on Asp^117^, Glu^221^, and Glu^224^ residues; the distance between the different residues is indicated in *yellow* (Å).

A model of LHCSR3 (Fig. 1) was created using as a template the three-dimensional structure of other LHC proteins, LHCII, CP29, and LHCI (39–41), with the aim of identifying protonatable residues exposed to the thylakoid lumen. The analysis of the protein model is consistent with LHCSR3 conserving the three trans-membrane α-helices (helix A, B, and C) and two amphipathic helices (helix D and E) revealed from crystallographic analysis of CP29 and LHCII; a short additional helix was predicted at the C-terminal domain, significantly more extended than in LHCII or CP29. LHCSR3 bears several acidic amino acid residues (*i.e.* aspartate and glutamate) predicted to face the thylakoid lumen. In particular, the C-terminal domain contains eight acidic residues: Glu^231^, Glu^233^, Glu^237^, Asp^239^, Asp^240^, Glu^242^, Asp^244^, and Asp^254^, as described in a previous report (27). Additional residues exposed to the lumen include Glu^221^, Glu^224^ at the lumenal end of Helix D, the residues Asp^109^ and Asp^117^ in the loop between Helix B and Helix E, and residue Glu^218^, located in the loop connecting Helix A and Helix D. To evaluate the accessibility of these residues to the solvent, the LHCSR3 protein sequence was analyzed by I-TASSER software (30, 31), which ranks the probability of a residue to be solvent-exposed within a range of 1 to 9, with a higher number indicating a higher probability. As reported in Table 1, the values obtained for most of the selected glutamates and aspartates were in the range of 3–4 with the exception of Glu^233^ and Glu^237^ (scoring 2) and Asp^254^ (scoring 6).

**TABLE 1:**
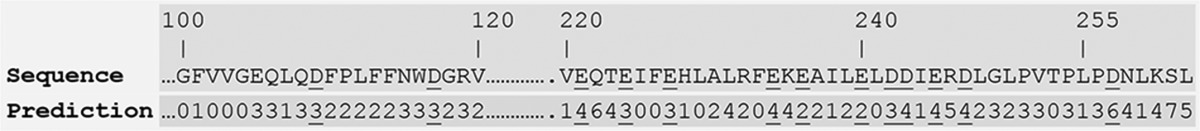
**Evaluation of the accessibility to the solvent of LHCSR3 lumen-exposed residues** Solvent accessibility was predicted using I-TASSER software which scores each protein residue with a 0–9 figure with high score indicating higher probability for solvent exposure. Lumen-exposed regions are reported together with the solvent-accessibility score for each residue shown below. Amino acid position, within the LHCSR3 sequence is indicated above.

##### Identification of the LHCSR3 Protonatable Sites Involved in Lumenal pH Sensing

**FIGURE 2.**
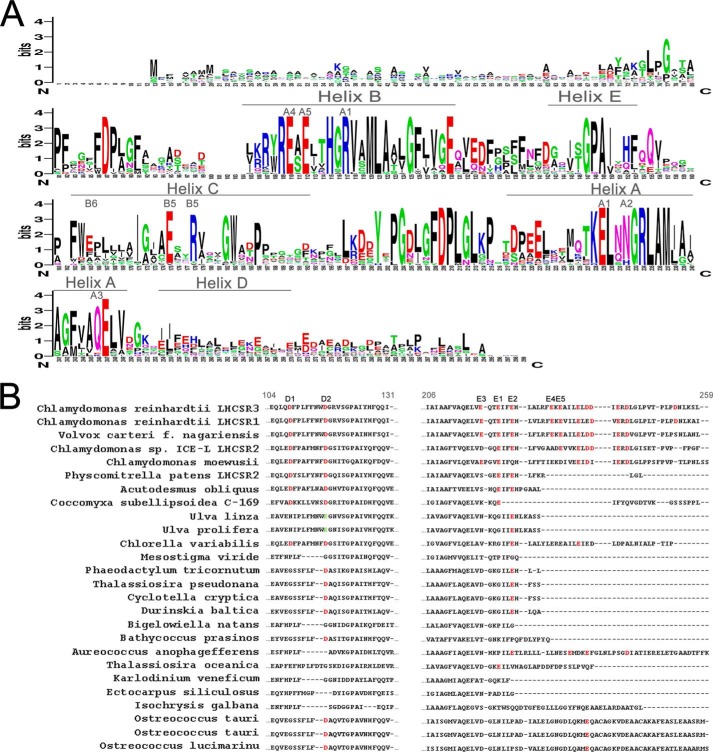
**Alignment of LHCSR-like protein sequences.**

The evaluation of the conservation of specific residues in sequences from different species can facilitate the identification of residues that are crucial for protein function. When the LHCSR3 protein sequence from *C. reinhardtii* was compared with homologous sequences from other organisms, either microalgae or mosses (Table 2), several residues appeared to be highly conserved (Fig. 2*A*). Most of the chlorophyll-binding sites previously identified in LHCSR3 from *C. reinhardtii* can also be found in LHCSR proteins from other species, namely the residues binding chlorophyll at the A1, A4, and A5 sites, according to the nomenclature previously used for LHCII chlorophyll binding sites (18, 42). However, some variability can be found for the A2, B5, and A3 sites. Finally, a residue at the position of binding site B6 (glutamate) was found only in a few sequences. As for the lumen-exposed residues from *C. reinhardtii*, their conservation is far from complete among the different sequences. In particular, as reported in Fig. 2*B*, the C-terminal domain, which was reported to be the knob of a dimmer switch to control the transition to a dissipative state (27), is present only in *Chlamydomonas* species, *Volvox carteri*, and *Aureococcus anophagefferens,* whereas the number of acidic residues within this domain is variable in the different organisms (Fig. 2*B*). LHCSR-like sequences from *Ostreococcus tauri, Ostreococcus lucimarinus*, and *Chlorella variabilis* show a single protonatable glutamate residue in the C terminus. Moreover, this domain was significantly shorter in the remaining sequences. In contrast, the residues in Helix E, Glu^221^ or Glu^224^ in LHCSR3 from *C. reinhardtii*, were conserved in 18 of 26 sequences analyzed. As for residue Glu^218^, this is only found in *C. reinhardtii*, *Chlamydomonas moewusii,* and *V. carteri*, whereas in *Chlamydomonas* sp. *ICE* a conservative replacement to aspartate was identified. Nevertheless, a glutamate was found very close to this position and shifted toward the N terminus in all the other accessions, suggesting it might have a conserved functional role. The analysis of the conservation of residues Asp^109^ and Asp^117^ showed that Asp^109^ is present in 9 accessions, whereas Asp^117^ is present in 17 accessions as aspartate, or replaced by glutamate in sequences from *Ulva linza* and *Ulva prolifera*. It is worth noting, however, that all the accessions that do not bear Asp^117^, do have one or more aspartate residues within 1–3 positions, the only exception being the sequence from *Mesostigma viride* lacking aspartates or glutamates in that protein domain. On the basis of these results, Asp^109^, Asp^117^, Glu^218^, Glu^221^, Glu^224^, Glu^231^, and Glu^233^ were selected for further investigation and renamed, respectively, D1, D2, E3, E1, E2, E4, and E5 for simplicity.

**TABLE 2:** **LHCSR-like protein sequences used for the determination of conserved residues** Protein sequences were selected by BLAST search using LHCSR3 mature protein sequence as query. Each sequence was selected for having a score >150 and *e*-value <6 *e*^−41^.

Protein sequence	Organism
XP_001696064.1	*C. reinhardtii* LHCSR3
XP_001696125.1	*C. reinhardtii* LHCSR1
XP_002948670.1	*V. carteri f. nagariensis*
ADP89594.1	*Chlamydomonas* sp. ICE-L LHCSR2
Q03965.1	*C. moewusii*
XP_001768071.1	*P. patens* LHCSR2
ABD58893.1	*Acutodesmus obliquus*
XP_005647960.1	*Coccomyxa subellipsoidea* C-169
ADY38581.1	*U. linza*
ADU04518.1	*U. prolifera*
XP_005848576.1	*C. variabilis*
ABD37894.1	*M. viride*
XP_002178699.1	*Phaeodactylum tricornutum*
XP_002295258.1	*Thalassiosira pseudonana*
CAA04403.1	*Cyclotella cryptica*
AHH80644.1	*Durinskia baltica*
DAA05890.1	*Bigelowiella natans*
CCO66741.1	*Bathycoccus prasinos*
EGB07306.1	*A. anophagefferens*
EJK65083.1	*Thalassiosira oceanica*
ABV22207.1	*Karlodinium veneficum*
CBJ27803.1	*Ectocarpus siliculosus*
ABA55525.1	*Isochrysis galbana*
XP_003079276.1	*O. tauri*
AAY27550.1	*O. tauri*
XP_001417976.1	*O. lucimarinus*

##### In Vivo Mutation Analysis

**FIGURE 3.**
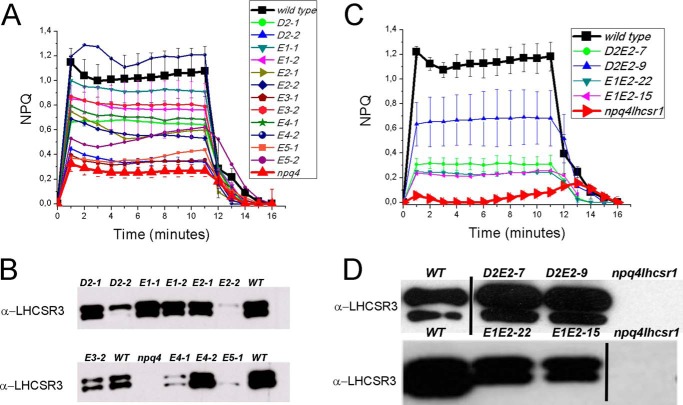
**NPQ measurements and immunoblot analysis of LHCSR3 protein levels in *npq4* lines expressing site-specific mutant versions of LHCSR3 affecting protonatable sites.** *Panel A,* NPQ measurements on WT, *npq4* mutant, and transgenic lines with LHCSR3 proteins carrying a single mutation on putative protonatable sites D2, E1–5. *Panel B,* immunoblot analysis of LHCSR3 accumulation on genotypes analyzed in *panel A*; immunoblot analysis of the D1 subunit of PSII is shown as a control for loading. *Panel C,* NPQ measurements on WT, *npq4 lhcsr1* mutant, and transgenic lines with LHCSR3 proteins with double mutations on putative protonatable sites D2E2 and E1E2. *Panel D,* immunoblot analysis of LHCSR3 accumulation on genotypes analyzed in *panel C*. In all cases three independent biological replicates were analyzed. The experiments were reproduced two times.

To test the importance of these protonatable residues for LHCSR3 function, each of the selected aspartate or glutamate residues was mutagenized *in vitro* to asparagine or glutamine, respectively, and the resulting mutant *LHCSR3* genomic DNA sequence was used to transform the *npq4* mutant strain lacking LHCSR3 expression (13). After selection of transformants based on paromomycin resistance, these were screened to determine the effect of the amino acid replacement on the NPQ activity. Fig. 3*A* shows that when each individual acidic residue was mutated, the NPQ amplitude was reduced, but a significant level of quenching was still present. Indeed, the transformant lines exhibited an NPQ level proportional to the level of LHCSR3 protein accumulation as assessed by Western blotting (Fig. 3*B*), suggesting redundancy of the proton-sensing residues in LHCSR3. Strains transformed with LHCSR3 variants mutated at residue D1 did not show any accumulation of LHCSR3, suggesting a major role of this residue in stabilizing protein folding. Therefore, combinations of multiple mutations within the same protein were generated and tested. When D2, E1, and E2 residues were mutated together, the triple mutant had an NPQ amplitude similar to that of the *npq4* strain. Indeed, of more than 300 colonies of the triple mutant D2E1E2 in the *npq4* background, none had higher NPQ than *npq4*, despite the accumulation of the mutant LHCSR3 protein at wild-type level.

**FIGURE 4.**
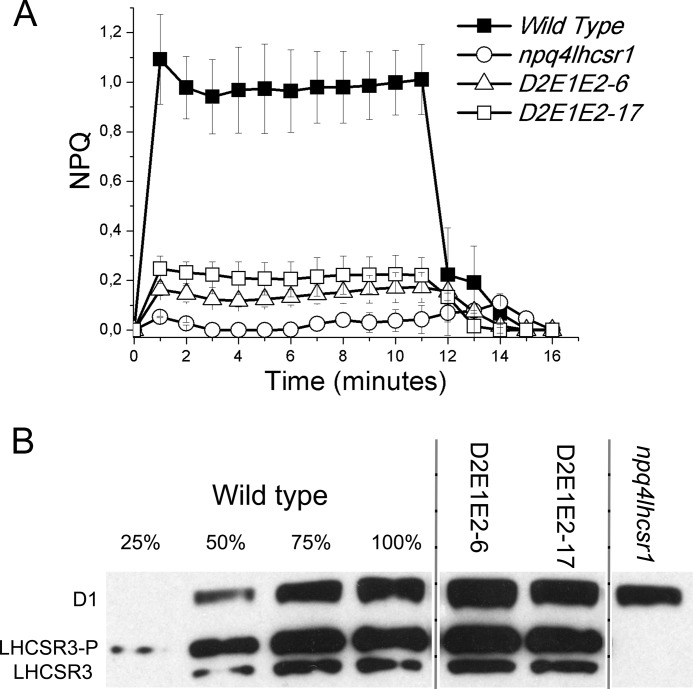
**NPQ measurements and immunoblot analysis of LHCSR3 protein levels in *npq4 lhcsr1* lines expressing site-specific mutant versions of LHCSR3 affecting protonatable sites.** NPQ measurements on WT, *npq4 lhcsr1* mutant, and transgenic lines with LHCSR3 proteins mutated on D2, E1, and E2 protonatable sites (*panel A*). *Panel B,* immunoblot analysis of LHCSR3 accumulation; immunoblot analysis of the D1 subunit of PSII is shown as a control for loading. In all cases three independent biological replicates were analyzed. The experiments were reproduced three times.

To improve the signal to background ratio in the NPQ assays, subsequent transformations were done with the *npq4 lhcsr1* double mutant (32). This system would allow for better resolution of the effect that the mutated LHCSR3 protein has on NPQ, independent of the LHCSR1 isoform that remains in the *npq4* mutant. Combinations of the double mutations, D2E2 and E1E2, were made and transformed into *npq4 lhcsr1* (Fig. 3, *C* and *D*). These double mutations impaired but did not completely eliminate the qE function of LHCSR3, because the expressed LHCSR3 protein still conferred some NPQ in the *npq4 lhcsr1* background (Fig. 3, *C* and *D*). Lines of the E1E2 mutant accumulating LHCSR3 at 50–60% with respect to the wild-type had ∼20% of wild-type qE (Fig. 3*C*). Two lines from the D2E2 transformations with more than wild-type LHCSR3, had only ∼30 and ∼50% of the qE found in wild-type, respectively (Fig. 3*C*). The D2E1E2 triple mutant version of LHCSR3 was then transformed into the *npq4 lhcsr1* genotype. As shown in Fig. 4, two independent lines expressed the D2E1E2 mutant protein at a level close to the wild-type LHCSR3 protein level, but they exhibited the lowest qE activity observed (∼25% of the wild-type level). The residual qE induction observed in the triple D2E1E2 mutant could be related to the activity of one or more of the other protonatable sites that are still present in the mutant. Unfortunately, the addition of further mutations in the *LHCSR3* gene resulted in a loss of protein accumulation, suggesting a strong destabilization of the protein or some impairment in protein import into the thylakoid membranes.

##### In Vitro Reconstitution of LHCSR3 Recombinant Protein Mutated on Protonatable Sites

The function of the three identified protonatable sites in LHCSR3 was next investigated *in vitro* using recombinant proteins. In particular, the *LHCSR3* cDNA sequence was subjected to site-specific mutagenesis of D2, E1, and E2, as previously described (43). Following expression in *E. coli* and purification, the wild-type (WT) and mutant D2E1E2 apoproteins were refolded *in vitro* in the presence of chlorophylls and carotenoids (18, 27, 33). As shown in Table 3, the holoproteins were characterized by HPLC pigment analysis. In both cases the Chl *a*/*b* ratio was higher than 8, with a very small amount of Chl *b* per apoprotein compared with Chl *a*. The Chl/Car ratio was also similar in WT and the D2E1E2 mutant with 2 Car molecules per 7 Chls bound in LHCSR3. The carotenoids bound by reconstituted samples were mainly violaxanthin and lutein in agreement with previous reports (18, 27).

**TABLE 3:** **Pigment analysis of recombinant LHCSR3 proteins** Pigment analysis were performed by HPLC and fitting of absorption spectrum of pigment acetone extracts with chlorophylls and carotenoids spectral forms as described in Ref. 34. The experiments were performed two times, with three independent biological replicates each time.

	Chl total	Chl *a*	Chl *b*	Viola	Lute	Neo	β-car	Chl/Car	Chl *a/b*	Car total
**WT**	7	6.29	0.71	0.41	1.56	0.06	0.00	3.38	8.86	2.07
**S.D.**	*/*	*0.10*	*0.05*	*0.03*	*0.07*	*0.01*	*0.00*	*0.31*	*0.64*	*0.19*
**D2E1E2**	7	6.51	0.49	0.28	1.63	0.00	0.00	3.61	13.29	1.94
**S.D.**	*/*	*0.09*	*0.07*	*0.03*	*0.05*	*0.01*	*0.00*	*0.22*	*1.91*	*0.11*

**FIGURE 5.**
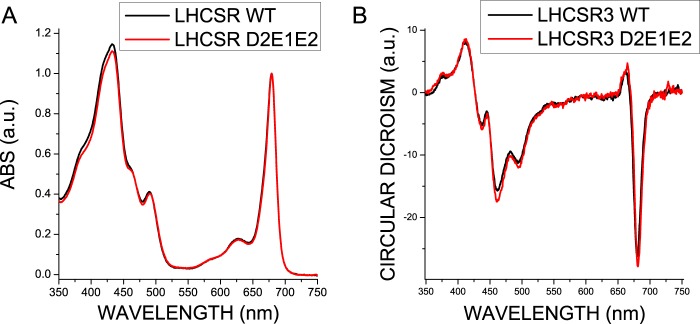
**Absorption and circular dichroism spectra of LHCSR recombinant proteins.** Absorption spectra (*panel A*) and circular dichroism (*panel B*) in the visible region of LHCSR3 WT and D2E1E2 mutant refolded *in vitro* in the presence of chlorophylls and carotenoids. The experiments were reproduced three times, each time with two independent biological replicates.

The efficiency of energy transfer between pigments was investigated by recording fluorescence emission spectra at room temperature upon selective excitation of Chl *a*, Chl *b*, and xanthophylls, showing no differences between WT and D2E1E2 proteins. The absorption spectra of WT and D2E1E2 in the visible region (Fig. 5*A*) did not show significant differences, neither in the Soret nor in the Qy spectral regions, suggesting that the mutations introduced no changes in the pigment organization in the complex. Similarly, circular dichroism spectra of the two samples were virtually identical (Fig. 5*B*). To assess if the mutations introduced could induce some level of protein destabilization, the thermal stability of recombinant WT and D2E1E2 mutant proteins was measured by following the change of the amplitude of the CD signal at 681 nm when slowly increasing the temperature of the samples. The melting temperatures (*T_m_*), calculated by fitting to a sigmoidal function, are reported in Table 4: *T_m_* was similar for WT and the D2E1E2 mutant, 41.8 and 40.2 °C, respectively, suggesting that substitution of the three acidic residues in the D2E1E2 mutant did not alter the stability of the pigment-protein complex.

**TABLE 4:** **Thermal stability of LHCSR WT and D2E1E2 recombinant proteins refolded *in vitro*** Thermal stability (*T_m_*) was evaluated following the decay of the CD signal at 682 nm when increasing the temperature from 20 to 80 °C. The thermal stability of the samples was determined by finding the *T*½ of the signal decay. The experiments were performed two times, with two independent biological replicates each time.

	*T_m_*	S.D.
	°*C*	
**WT**	41.8	2.3
**D2E1E2**	40.2	0.9

##### [^14^C]DCCD Binding of Recombinant LHCSR3 Proteins

**FIGURE 6.**
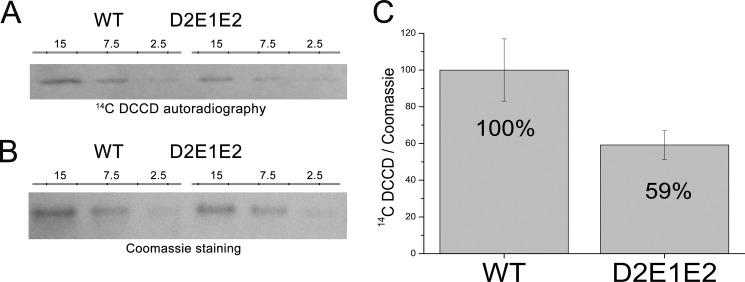
**^14^DCCD binding in LHCSR3 recombinant WT and D2E1E2 mutant.** *Panel A,* autoradiography of recombinant LHCSR3 WT and the D2E1E2 mutant treated with ^14^DCCD; microliters of sample (0.2 μg/μl of chlorophylls) loaded on SDS-PAGE are reported (15, 7.5, and 2.5 μl). *Panel B,* Coomassie staining of SDS-PAGE used for autoradiography. *Panel C,* ratio of the level of ^14^C observed by autoradiography signals and protein quantity obtained by densitometric analysis of Coomassie-stained gels. The experiments were performed two times; each time two independent biological replicates were analyzed with three technical replicates with different loading volume as indicated.

DCCD is an inhibitor of qE in *Chlamydomonas* (18). Its binding to acidic residues indicates reversible protonation events. An enhanced DCCD binding with respect to other LHC proteins has been reported for LHCSR3 (18), in agreement with its pH-sensing function. To assess the proton-binding activity of the Asp^117^, Glu^221^, and Glu^224^ residues, DCCD binding was measured in WT and D2E1E2 mutant proteins. *In vitro* refolded proteins were incubated with [^14^C]DCCD, and the amount of ^14^C bound by LHCSR3 was determined by autoradiography. The level of ^14^C bound by LHCSR3 WT and D2E1E2 was then normalized to the protein amount loaded into the SDS-PAGE gel quantified by Coomassie staining (Fig. 6). As reported in Fig. 6, both WT and D2E1E2 mutant bound [^14^C]DCCD, but binding to the D2E1E2 mutant was decreased by 40% with respect to WT. This result supports the hypothesis of multiple protonatable sites in LHCSR3, of which Asp^117^, Glu^221^, and Glu^224^ account for at least 40% of the DCCD-binding activity of this protein.

##### Fluorescence Lifetimes of Recombinant LHCSR3 Proteins

**FIGURE 7.**
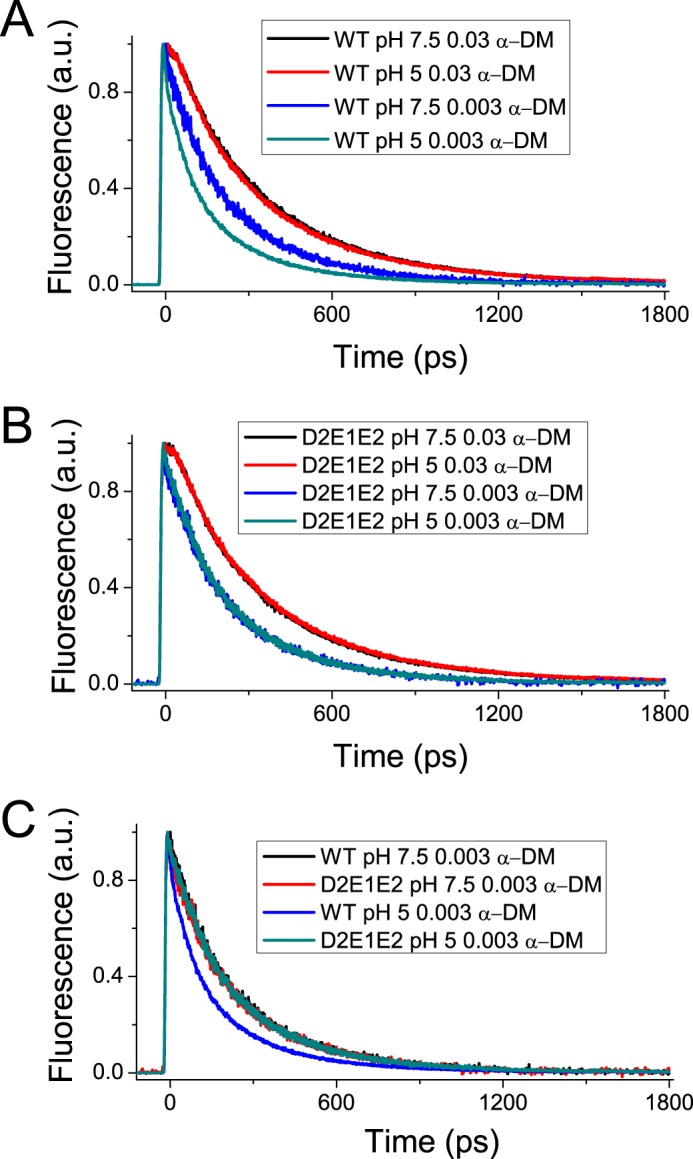
**Fluorescence decay kinetics.** Fluorescence decay kinetics of recombinant LHCSR3 WT (*panel A*) and D2E1E2 mutant (*panel B*) at pH 7.5 or 5.0 in the presence of high (0.03%) or low (0.003%) detergent (α-DM) concentrations. The experiment was performed two times, each time with two independent biological replicates.

Fluorescence lifetime measurements on recombinant LHC proteins allow investigation of their excitation energy conserving *versus* quenching properties (44). To investigate *in vitro* the pH-dependent regulation of LHCSR3 quenching activity, fluorescence decay kinetics of WT LHCSR3 and the D2E1E2 mutant were measured using a single photon counting device at neutral pH (7.5) and at low pH (5) in detergent solution of 0.03% *n*-dodecyl-α-d-maltopyranoside (α-DM). As reported in Fig. 7*A*, the fluorescence decay kinetics of WT and the D2E1E2 mutant can be satisfactorily fitted with three exponentials with associated time constants of 4 ns, 1.9 ns, and ∼200 ps. The relative amplitudes were 38–44, 48–52, and 7.4–9.7%, respectively, with an average lifetime of 2.6–2.7 ns (Table 5). Decays were similar at both pH 7.5 and 5, suggesting no pH-dependent response of quenching reactions.

**TABLE 5:** **Fluorescence lifetimes of LHCSR3 WT and D2E1E2 mutant** The decay traces reported at Fig. 7 were fitting using three exponentials functions. The amplitude (A) and time constants (τ) for each exponential are reported in the table. The average lifetimes for each sample are calculated as ΣA_i_τ_i_.

	A1	τ1	A2	τ2	A3	τ3	τ_avg_
		*ns*		*ns*		*ns*	
**WT, pH 7.5, 0.03% α-DM**	44%	4.04	48%	1.88	8%	0.21	2.69
**WT, pH 5, 0.03% α-DM**	40%	4.22	51%	1.91	10%	0.19	2.71
**D2E1E2, pH 7.5, 0.03% α-DM**	36%	4.37	54%	1.93	10%	0.18	2.63
**D2E1E2, pH 5, 0.03% α-DM**	39%	4.20	52%	1.83	9%	0.23	2.61
**WT, pH 7.5, 0.003% α-DM**	40%	2.61	31%	1.00	29%	0.14	1.39
**D2E1E2, pH 7.5, 0.003% α-DM**	41%	2.65	30%	0.98	29%	0.14	1.42
**WT, pH 5, 0.003% α-DM**	25%	2.50	33%	0.84	42%	0.14	0.97
**D2E1E2, pH 5, 0.003% α-DM**	41%	2.63	30%	0.99	29%	0.14	1.41

This result is in agreement with a previous report on LHCSR3 fluorescence lifetime in detergent (27), suggesting that interaction of detergent micelles with the protein prevents the switch to a dissipative conformation. pH sensitivity of the LHCSR fluorescence lifetime can be better detected at a low detergent/protein ratio leading to moderate aggregation, which reproduces protein-protein interactions occurring in the protein-crowded thylakoid membrane (45). Fig. 7*B* shows the fluorescence lifetimes of recombinant WT LHCSR3 and the D2E1E2 mutant as measured upon incubation in a detergent concentration of 0.003% α-DM. These measuring conditions induced a faster decay of emitted fluorescence at either pH 5 or 7.5 for both LHCSR3 WT and D2E1E2 mutant. However, whereas at pH 7.5 the two proteins showed the same decay profile, at pH 5 LHCSR3 WT fluorescence decay was much faster than at pH 7.5, whereas the fluorescence decay of LHCSR3 D2E1E2 was the same as at pH 7.5. Decays of LHCSR3 WT at pH 7.5, and D2E1E2 at pH 7.5 and 5 were fitted to three exponentials with time constants of 2.5 ns, 0.9 ns, and 140 ps with amplitudes of 40, 31, and 29%, respectively, with an average lifetime of ∼1.4 ns (Table 5). LHCSR3 WT decay traces at pH 5 were similarly fitted to three exponentials, but in this case the major amplitude was associated to the fastest component (140 ps) with amplitude of 42%, whereas those with 0.8 and 2.5 ns showed amplitudes of 33 and 25%, respectively (Table 5). Because aggregation is well known to influence the lifetime of LHC proteins (46), the aggregation size of WT and D2E1E2 proteins at 0.03 and 0.003% DM was measured by dynamic light scattering as previously reported (38), yielding the average aggregate size (113.3 ± 8.6 in the case of WT and 126.9 ± 18.2). LHCSR3 WT and D2E1E2 in low detergent condition at pH 5 formed aggregates with similar size with a radius of 100 nm, implying that the difference in fluorescence quenching observed between WT and D2E1E2 is likely due to the different dissipative conformations that can be reached by the two proteins.

##### Fluorescence Emission Spectra at 77 K

**FIGURE 8.**
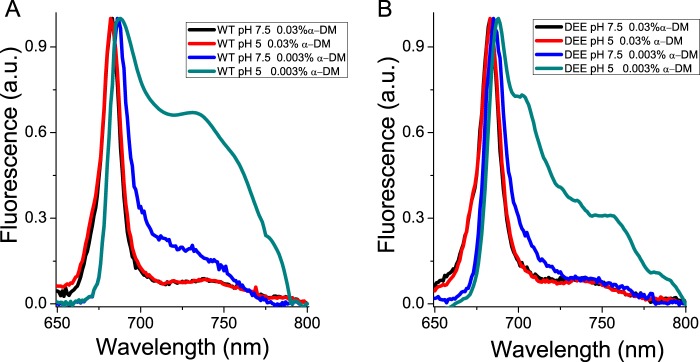
**77 K fluorescence emission spectra.** Fluorescence emission spectra of recombinant LHCSR3 WT (*panel A*) and the D2E1E2 mutant (*panel B*) at 77 K measured at pH 5 or 7.5 in the presence of 0.03% α-DM or 0.003% α-DM. The experiments were reproduced three times, each time with two independent biological replicates.

Activation of quenching mechanisms has been previously associated *in vivo* with induction of far-red fluorescence emission forms at 77 K (38, 47, 48). In particular, aggregation-dependent quenching in LHC proteins at low pH was shown to lead to far-red emission in both trimeric and monomeric isoforms, and this feature has been correlated with the extent of excitation energy quenching (38). The fluorescence emission spectra at 77 K of LHCSR3 WT and D2E1E2 recombinant proteins were measured to investigate the correlation of far-red emission forms with the activation of quenching mechanisms. The measurements were performed at high (0.03%) or low (0.003%) detergent concentrations and at pH 7.5 or 5.0. As reported in Fig. 8, fluorescence emission spectra were almost identical at high detergent conditions for both WT and D2E1E2 samples at pH 7.5 or 5 with a peak at 682 nm. At low detergent, instead, a clear shift of the emission peak to 685 or 687 nm was observed for D2E1E2 and WT, respectively. The most evident change in the spectra, however, was observed at low detergent concentrations and pH 5, where both WT and D2E1E2 dramatically increased their far-red emission forms, with the formation of a defined peak at 735 nm that was far more intense in WT *versus* D2E1E2. These results support the presence of a positive relationship between protonation of specific residues, the appearance of far-red emission forms in the spectra, and the activation of quenching mechanisms in LHCSR3.

## Discussion

All oxygenic photosynthetic organisms are endowed with mechanisms for thermal dissipation of excess absorbed light energy. The triggering of these mechanisms can either be controlled directly by light as for the Orange Carotenoid-binding protein of cyanobacteria (49, 50) or by low lumenal pH caused by excess light as in the case of PSBS in plants and LHCSR in unicellular algae (10, 13, 18, 26, 51, 52). LHCSR3 is of particular interest because the quenching and the pH-sensing activities are merged in the same protein subunit (18, 27, 53) making this protein a relatively simple system for the molecular analysis of NPQ. The case of plants is more complex because the pH is sensed by PSBS, whereas quenching occurs in an interacting pigment-binding partner (17, 26). LHCSR3 has been reported to undergo functional changes depending on pH (18, 27, 53). In this work, the function of LHCSR3 as a sensor of lumen pH has been investigated *in vivo* by site-specific mutagenesis of putative protonatable residues. The LHCSR3 structure was modeled on the basis of LHCII and CP29 structures (39, 40) (Fig. 1) allowing for identification of 13 potentially protonatable aspartate and glutamate residues located within lumen-exposed domains at the C terminus, at Helices D and E, and at the loops between Helices A and D and between Helices B and E. Among these residues, Asp^117^, Glu^218^, Glu^221^, Glu^224^, Glu^231^, and Glu^233^ were selected based on their high conservation among LHCSR-like sequences and were targeted for site-specific mutagenesis and functional analysis *in vivo*. The complementation of *npq4* and *npq4 lhcsr1* mutants with sequences carrying mutations affecting these protonatable residues identified Asp^117^, Glu^221^, and Glu^224^ as the key residues for the pH sensitivity of LHCSR3 *in vivo*. Any single mutation of an acidic amino acid residue failed to yield significant effects on qE. When double mutants affecting two different putative protonatable sites were obtained, a substantial decrease in qE relative to the LHCSR3 protein level could be observed, suggesting a cooperative behavior (Fig. 4). The triple D2E1E2 mutant expressed in *npq4 lhcsr1* showed an even greater impairment of function, suggesting that Asp^117^, Glu^221^, and Glu^224^ are key residues for pH sensing in LHCSR3 from *C. reinhardtii*.

These results are consistent with the significant decrease observed in DCCD binding to LHCSR3 recombinant proteins mutated on Asp^117^, Glu^221^, and Glu^224^, *i.e.* a reduction by 41% (Fig. 6). The fact that DCCD can still be bound by the D2E1E2 mutant is not surprising, because structure modeling revealed the presence of 10 additional acidic residues, including 8 glutamate and aspartate residues at the C terminus that are likely to bind DCCD in the D2E1E2 mutant. It is interesting to note that the mutation of 23% of the putative protonatable residues, as in the case of D2E1E2 mutant, led to a 41% reduction in DCCD-binding activity of LHCSR3, suggesting that these residues have a special cooperative role in transducing the lumenal pH signal, as shown by a 72% of reduction in qE *in vivo* (Fig. 4). The presence of additional glutamate and aspartate residues at the C terminus in LHCSR3 is a peculiar feature of *Chlamydomonas* spp. LHCSR proteins (Fig. 2), whereas other LHCSR-like proteins have a shorter C terminus extension (Fig. 2*B*). Asp^117^, Glu^221^, and Glu^224^ are more conserved in the different LHCSR-like sequences analyzed. Nevertheless, it is worth noting that a substantial level of variability is present in the position and number of acidic residues, which might reflect the need for complementarity with interacting proteins putatively involved as partners in qE activity (54, 55). Alternatively, the density of lumen-exposed acidic residues might be related to the response sensitivity for triggering qE, which is particularly strong in *Chlamydomonas*, for which light saturation of photosynthesis occurs at lower irradiances (56), whereas NPQ amplitude is fully reached already at 200 μmol m^−2^ s^−1^ illumination (57).

It is interesting to compare the distribution of acidic residues in *Chlamydomonas* to that of *Physcomitrella patens* LHCSR1, which only harbors 4 of the 13 putative protonatable residues identified in LHCSR3. *P. patens* LHCSR1 has been shown to require zeaxanthin for a significant level of activity (25), whereas *Chlamydomonas* NPQ is not dependent on zeaxanthin accumulation (18). Zeaxanthin binding has been shown to confer cooperativity to NPQ in higher plants (58–60). It is tempting to propose that zeaxanthin might replace the effect of the 9 additional lumen-exposed acidic residues in promoting the switch of LHCSR from conservative to dissipative conformations. Indeed, we verified that faster fluorescence decay was triggered by acidic pH in WT LHCSR3 but not in the D2E1E2 mutant, implying that the protonation of Asp^117^, Glu^221^, and Glu^224^ has a special role in triggering quenching events within LHCSR3 *in vitro* (Fig. 7). A previous report has shown that mutation of 9 acidic residues at the C terminus, including Glu^224^, led to impaired pH sensitivity of LHCSR3 *in vitro* (27). Here we show that 72% of qE activity *in vivo* was dependent on the mutation of only three protonatable residues, consistent with loss of pH responsiveness *in vitro*. Residual NPQ activity in D2E1E2 is likely due to the presence of several other protonatable residues in the D2E1E2 mutant, partially inducing a small NPQ activation in the triple mutant. By the way it could not be excluded that the protonation of other LHCBM subunits, as LHCBM1 (18), would contribute to pH-dependent triggering of a low NPQ activity in D2E1E2.

As previously reported, LHCSR3 does not respond significantly to pH variation when the protein is dissolved in detergent such as α-DM, whereas the pH sensitivity becomes evident when detergent is substituted by nano-polymers (27) or decreased to levels below the critical micelle concentration. This latter condition induces the formation of small particle arrays, mimicking protein-protein interactions in the thylakoid membrane (47), a condition likely to also occur in PSII-LHCSR supercomplexes (53). Recent results showed that high detergent conditions favor monodispersion of LHCs and shift their conformation far from the dissipative state toward a state poorly responding to pH variations (45). This is likely due to the induction of a relaxed protein conformation that decreases pigment-pigment interactions within the complexes with respect to the state present in the native membrane environment. Structural analysis suggested that conformational changes involved in quenching are subtle (61, 62) and involve small changes in Chl-Chl and xanthophyll-Chl interactions (11) thus making the relaxed structure unfavorable to trigger quenching. The main effect induced at pH 5 on fluorescence decay kinetics of WT LHCSR3 was an increased amplitude of the 140-ps (τ3) component, which favorably compares with the 65- and 305-ps components recently identified as induced *in vivo* upon qE activation in *C. reinhardtii* (10) and the 200-ps component identified when measuring fluorescence lifetimes of LHCSR3-binding PSII supercomplexes at pH 5 (53).

The mechanism by which LHCSR3 dissipates excitation energy quenching is still debated. High yield of a carotenoid radical cation has been previously reported (18), and formation of these radical species has been previously related to NPQ in plants (63, 64). Here, we present evidence that aggregation-dependent quenching is also active in LHCSR3, as in other LHC proteins (38, 45, 46, 59, 62, 65, 66). Interestingly, a strong red-shift in fluorescence emission was associated with the low pH effect in the LHCSR3 WT, but not in the D2E1E2 mutant (Fig. 5). The formation of these far-red emitting forms is dependent on pH and protonation of Asp^117^, Glu^221^, and Glu^224^. The correlation between far-red emission and switch to a dissipative state has been previously reported for plant LHCII (47), possibly resulting from a strong coupling between chlorophylls (67). It is interesting to note that the only Chl-binding residues fully conserved through LHCSR-like sequences are those associated with sites A1, A4, and A5 (Fig. 2), as putative ligands for Chl 601, Chl 610, and Chl 609 (39). Site A1 has been previously reported to be crucial for protein stability in most LHC proteins (34, 43) acting as a bridge between Helices A and B. Chl-binding sites A4 and A5, instead, are located in proximity of the Car-binding site L2, with a special role in ^3^Chl* quenching (68). Together with the Chl in site B5, these Chls form a strongly coupled cluster (40, 69), which has been associated to Car radical cation formation (11). Interactions between Chls and between Chls and xanthophylls are likely involved in LHCSR3 quenching activity. The structural model of LHCSR3 in Fig. 1 shows that the three acidic residues Asp^117^, Glu^221^, and Glu^224^ are relatively close to each other, with an estimated distance of 3.69, 7.27, and 8.05 Å, respectively (Asp^117^-Glu^221^, Glu^221^-Glu^224^, and Asp^117^-Glu^224^ in Fig. 1*B*). The proximity of these residues, shown to be crucial and cooperative in transducing pH sensing, suggests that upon their protonation the overall structure of LHCSR3 might undergo adjustments that reduce the distance/relative orientation between the helices as a result of reduced electrostatic repulsion in the lumen-exposed domain. In particular, a different distance between Helix D and Helix E might be induced by protonation of Asp^117^, Glu^221^, and Glu^224^. This could be transduced into changes in the relative orientation of Helices A and B, with a consequent reorganization of Chl-Chl and Chl-Car interactions. The correspondence between protein aggregation *in vitro* and NPQ activation *in vivo* has been previously investigated, showing a similarity between the conformational change induced *in vitro* by aggregation of LHC proteins and conformational changes observed *in vivo* upon NPQ induction (14). In addition, we cannot exclude protein aggregation *in vitro* forces LHCSR3 subunits to establish some peculiar protein-protein interactions required for LHCSR3 activity *in vivo*. LHCSR proteins can be found as dimers in thylakoid membranes (18, 70), suggesting that possibly some specific protein-protein interactions are needed for LHCSR3 activity. The finding of LHCSR proteins as dimers agrees with recent crystallization of PSBS at low pH in the dimeric state (71), suggesting a possible common strategy for protein activation by formation of homo- or heterodimers and rearrangements of PSII supercomplexes. However, it should be pointed out that whereas LHCSR3 is a chlorophyll- and carotenoid-binding protein, PSBS was reported to bind a single chlorophyll at most. These different pigment-binding properties suggest that whereas LHCSR3 can be a direct quencher of excitation energy located on its pigments, PSBS function is more likely restricted to pH sensing, whereas triggering quenching is activated within interacting LHC proteins. Finally, it is interesting to compare the effects of mutation on protonatable residues in LHCSR3 as compared with PSBS. Recently, the structure at low pH of PSBS from spinach was revealed (71), showing the DCCD binding site at residue Glu^173^. Previously it was shown indeed that in *A. thaliana* mutation of two PSBS lumen-exposed glutamate residues, Glu^122^ (corresponding to Glu^173^ in PSBS from spinach) and Glu^226^, yielded complete loss of qE *in vivo* and DCCD binding *in vitro*. The effect of individual mutations was additive, with changes to non-protonatable residues at each residue leading to 50% loss in both functions (17). This is clearly not the case for LHCSR, because no effect was observed upon mutation at single residues and even the D2E1E2 mutant still retained 28% of qE and 59% of DCCD binding. Thus, it appears that pH-dependent triggering is far more cooperative in LHCSR3 than in PSBS, with a number of contributing protonation events depending on species that have been reported to differ in the relative contribution by ΔpH and Δψ to the transmembrane pH gradient (72, 73). Also, responsiveness of different species to light intensity and adaptation to specific environments (15, 57) might be tuned by the number and distribution of lumen-exposed protonatable residues in LHCSR. These results are complementary to those recently reported (27) showing that pH responsiveness, as determined by fluorescence lifetime *in vitro*, was lost by mutation of 9 acidic residues at the C terminus (including Glu^224^, also studied in the present work) to non-protonatable species. The observed cooperativity between acidic residues might well explain this result.

## Author Contributions

K. K. N., R. B., and M. B. conceived the work, designed the experiments, and wrote the paper. K. K. N. coordinated the experiments about *Chlamydomonas* complementation (Figs. 3 and 4), whereas R. B. and G. F. coordinated the experiments *in vitro*. M. B. and G. R. S. performed all the experiments reported with the exception of *Chlamydomonas* complementation and mutant screening and characterization. T. B. T. and E. E. performed the work described in Figs. 3 and 4. G. R. F. and E. D. R. contributed to the results reported in Fig. 7. All authors analyzed the results, contributed to writing, and approved the final version of the manuscript.

## Footnotes

6 The abbreviations used are:

^3^Chl*: chlorophyll triplets
NPQ: non-photochemical quenching
α-DM: *n*-dodecyl-α-d-maltopyranoside
Car: carotenoid
Chl: chlorophyll
qE: energy-dependent quenching
LHCSR: light harvesting complex stress-related
DCCD: dicyclohexylcarbodiimide
PSBS: photosystem II subunit S.
